# ROS-Mediated Necrosis by Glycolipid Biosurfactants on Lung, Breast, and Skin Melanoma Cells

**DOI:** 10.3389/fonc.2021.622470

**Published:** 2021-03-16

**Authors:** Farazul Haque, Mohd Sajjad Ahmad Khan, Naif AlQurashi

**Affiliations:** ^1^Department of Immunology and Genomic Medicine, Graduate School of Medicine, Kyoto University, Kyoto, Japan; ^2^Department of Basic Sciences, Deanship of Preparatory Year and Supporting Studies, Imam Abdulrahman Bin Faisal University, Dammam, Saudi Arabia

**Keywords:** cancer, glycolipids, mitochondria membrane potential, necrosis, reactive oxygen species, synergy

## Abstract

Cancer is one of the major leading causes of death worldwide. Designing the new anticancer drugs is remained a challenging task due to ensure complexicity of cancer etiology and continuosly emerging drug resistance. Glycolipid biosurfactants are known to possess various biological activities including antimicrobial, anticancer and antiviral properties. In the present study, we sought to decipher the mechanism of action of the glycolipids (lactonic-sophorolipd, acidic-sophorolipid, glucolipid, and bolalipid) against cancer cells using lung cancer cell line (A549), breast cancer cell line (MDA-MB 231), and mouse skin melanoma cell line (B16F10). Scratch assay and fluorescence microscopy revealed that glycolipids inhibit tumorous cell migration possibly by inhibiting actin filaments. Fluorescence activated cell sorter (FACS) analysis exhibited that lactonic sophorolipid and glucolipid both induced the reactive oxygen species, altered the mitochondrial membrane potential (ΔΨ) and finally led to the cell death by necrosis. Furthermore, combinatorial effect of lactonic-sophorolipd and glucolipid demonstrated synergistic interaction on A549 cell line whereas additive effect on MDA-MB 231 and B16F10 cell lines. Our study has highlighted that lactonic-sophorolipd and glucolipid could be useful for developing new anticancer drugs either alone or in combination.

## Introduction

Surfactants are the compounds that alter the surface and interfacial tension at air/liquid or liquid/liquid interfaces. They are broadly devided into two categories, i.e., chemical and biological. Chemically derived surfactants play a crucial role in our daily life, although, these surfactants have several drawbacks over biologically derived surfactants or biosurfactants (BSs) (1). BSs have gained special interest due to their superior quality and potentialities to overcome the disadvantages of chemical surfactants. BSs are a group of diverse amphiphilic compounds and could be widely used in cosmetics, pharmaceuticals, food industries, and bioremediation (2). The most biologically active class of BSs are glycolipids (3). The glycolipids have gained focus by researchers due to their superior pharmaceuticals properties. The glycolipids (viz. sophorolipid, rhamnolipid etc.), like other BSs, can be easily produced by using cheap agri-wastes, used oil from restaurants or animal fats using fermentation processes. Glycolipids have become a possible alternative to be effectively utilized in pharmaceutical, food, biomedical, and health industries due to its exceptional biological activities such as antimicrobial, antiviral, anticarcinogenic, and immne-modulating potentials (4).

BSs like sophorolipid (SL), rhamnolipid, trehalolipid, and mannosylerythritol lipid (MEL) are the most important glycolipids found in nature. MEL is known for its antimicrobial, immunological, and neurological activities (5–7). Moreover, MEL has shown chromatin condensation, DNA fragmentation, cell cycle arrest, and production of melanin in B16F10 mouse melanoma cells which leads to apoptosis or cell death (8). The SL produced by *Wickerhamiella domercqiae* was shown to induce apoptosis in H7402 human liver cancer cells by blocking the cell cycle at G1 phase, activating caspase-3, and increasing Ca^2+^ concentration in the cytoplasm (9). The effects of different lengths of sophorolipid moleculeson human esophageal and pancreatic cancer cell lines, were also studied (10, 11). Glycolipid from *Sphingobacterium detergens* showed anticancer activity against Caco2 colorectal cancer cell line (12). On the other hand, trehalose lipid, a glycolipid from *Mycobacteria* is useful in the bioremediation and also act as an antitumor agent (13). Recently, our lab also showed that lactonic SL has antifungal activity by ROS generation and ultimately cell death by necrosis (14, 15). Whereas, rhamnolipid has shown antimicrobial activity alone or in combination with nisin (16). Nevertheless, these effects were not deeply investigated as many researchers concluded their results based on single cell line without using proper controls (non-cancer cell lines) (17).

Cancer is the second leading cause of death globally, accounting for an estimated 9.6 million deaths. Lung, prostate, colorectal, stomach, and liver cancers are the most common types of cancer in men, whereas breast, colorectal, lung, cervical, and thyroid cancers are the most common among women (18). Cancer of lung, liver, breast, stomach, and bowel are the most common cause of death worldwide. Lung cancer related death is ranking at the top followed by breast cancer (http://www.cancerresearchuk.org). Globally, 1.38 million new cases of breast cancer are diagnosed every year. Around 458,000 death from breast cancer occures per year worldwide, making it the most common cause of female cancer death in both developed and developing world (19). The reasons for the increased death rate are non-specific targets of anticancer molecules that causes severe side effects, and low cure rates (20) as well as resistance attained by cancer cells (21).

Considering the current scenario it is urgently required to develop highly specific and less toxic molecules for the anticancer therapy. Therefore, in our present study, we investigated anti-cancer efficacy of four different types of glycolipids, viz. acidic SL, lactonic SL, glucolipid, and bolalipid on three different cancer cell lines, i.e., lung (A549), breast (MDA-MB231), and mouse melanoma cell line (B16F10). We attempted to explore the mechanism of action of these glycolipids. Moreover, combinatorial effects between potential glycolipids were also evaluated. In our knowledge, this is the first report exhibiting the detailed mechanistic view of glycolipids on cancer cell lines as well as combinatorial effects when used in combination. This study could be useful in developing glycolipids as newer anticancer drugs.

## Materials and Methods

### Biosurfactants, Cell Lines, and Media

Both acidic and lactonic forms of SL were produced by our lab standardized protocol (7, 15). Whereas, bolalipid and glucolipids were synthesized by previously published protocols as adapted by Serens et al. (22) and Delbeke et al. (23). The human lung adenocarcinoma epithelial cell line A549, breast cancer cell line MDA-MB231 and mouse skin melanoma cell line B16F10 as well as normal human fibroblast cells MRC-5 were purchased from ATCC (American Type Culture Collection). A549 cells were maintained in DMEM (sigma) supplemented with 10% fetal bovine serum (FBS) (Sigma), 100 IU/ml penicillin and 100 µg/ml streptomycin in humidified 5% CO_2_ at 37°C. MDA-MB231 cells were cultured in DMEM supplemented with 6 mM l-glutamine, 20 mM Hepes, 10 μg/ml human insulin, 10% (vol/vol) FBS, 100 units/ml penicillin and 100 μg/ml streptomycin at 37°C, in 5% CO_2_. B16F10 cell line was cultured and maintained in Dulbecco’s-modified Eagle’s medium (DMEM) (Sigma) supplemented with 10% fetal bovine serum (FBS), and incubated at 37°C in a 95% O_2_ atmosphere.

### Cytotoxicity/MTT Assay

The cytotoxic effects of glycolipids were assessed against the cancer cell lines by MTT [3-(4,5-dimethylthiazol-2-yl)-2,5-diphenyltetrazolium bromide] assay. Briefly, 15 × 10^3^ cells of cell line suspensions per well of a 96-well plate were seeded and allowed to adhere for 12–16 h. Varying concentrations (0–1,000 µg/ml) of test glycolipids were made in Dimethylsulfoxide (DMSO). The concentration were made such that the final amount of DMSO should not exceed 0.2% and later added to the wells. After incubation for 24 h and 48 h, cell viability was detrmined by using MTT. A solution containing 10 µl of MTT and 90 µl of media was added to each wells and the cells were incubated for another 3 h at 37°C with 5% CO_2_. Later, solution containing media and MTT were taken out and 100 µl of DMSO (100%) was added to each well and mixed properly to dissolve the purple formazon crystals. The dissolved crystals were measured by spectrophotometry at 570 nm using microplate reader (Multiskan MK3, Thermo Lab systems). The cytotoxicity of glycolipids were expressed as a value of IC_50_ (The concentrations where 50% of the cell are dead). The effect of glycolipids on non-cancerous fibroblast cells (MRC-5) was also evaluated. Etoposide 10 nM (Sigma) used as a positive control.

### *In Vitro* Scratch Assay/Migration Assay

*In vitro* cell migration assay was performed in 12 well plates using a scratch method as adapted by Liang et al. (24). Briefly, 1 × 10^5^ cells of cell line suspensions were seeded in 12 well plates and allowed to adhere at 37°C with 5% CO_2_ until the complete monolayer is formed. Scratch was inflicted using the sterile toothpick. The medium was carefully removed and cells were washed three times with PBS. Further, 700 µl of serum free media with glycolipids or control (0.2% DMSO dissolved in media) was added. At 0 h, 24 h, and 48 h, wound were observed and photographed. Relative migration area, expressed in percentage, was obtained from equation 1 where, A_t_ represents the final cell free area, and A_to_ stands for initial wound area.

(1) $$RT=A_{t}\times 100/A_{to}$$

### Immunofluorescence Assay

All three cancer cells were seeded on sterilized lysine coated cover slips (lodged in a 12 well plate) in the number of 1 × 10^5^ cells, in their appropriate complete growth medium. Cells were allowed to attach for 12 h at 37˚C until it forms a complete monolayer on the coverslip. After 24 h of glycolipids treatment, cells were washed with PBS. Later, formalin was added to each well for 20 min for proper fixation. Subsequently, formalin is aspirated out and cells were washed with PBS three times, followed by blocking with 1% bovine serum albumin (BSA) at room temperature for next 1 h. Next, cells were stained with FITC-phalloidin antibody (1:100 v/v) and incubated at 4˚C overnight. Then, after washing with PBS three times, cells were counterstained with 4′,6-diamidino-2-phenylindole (DAPI) (1:1000 v/v) for 10 min. Phalloidin has a strong affinity toward actin filaments. Phalloidin tagged with fluorescent dye binds to actin and appears red in color whereas DAPI stained the nucleus and appears blue. Slides were prepared by mounting the coverslips in histamount (Sigma) and observed under Zeiss fluoresecence microscope (100× magnification) to observe the distribution of F-actin. Etoposide (10 nM) was used as positive control.

### Reactive Oxygen Species (ROS) Measurement

To determine baseline intracellular ROS levels, approximately 5 × 10^4^ cells were seeded in each well of 24 well plates and incubated at 37˚C, with 5% CO_2_ for 24 h. Later, media was aspirated out and cells were washed with PBS three times, and fresh medium containing various concentrations of glycolipids was added and plates were incubated at 37˚C for 3 h (DMSO 0.2% was used as vehicle control). After incubation, media was gently removed and ROS-specific dye H_2_-DCFDA (10 µM in PBS) was added for 10 min and incubated for 15 min at 37˚C. Next, dye was removed and cells were washed and 0.1% of trpysin solution in PBS was added. Finally, the cells were collected and acquired using flow cytometer (Beckman coulter, Gallios FACS).

### Mitochondria Membrane Potential (MMP) Measurement

The change of mitochondrial membrane potential was determined by the retention of the dye 3, 3′-Dihexyloxacarbocyanine iodide (DiOC_6_) using the method of Liu et al. (25). Briefly, 15 × 10^3^ cells were seeded in each well of the 96-well plate overnight prior to the experiments. Next day, cells were treated with different concentrations of glycolipids for 48 h. After treatment, cells were harvested, washed twice with PBS and stained with 50 nM DiOC_6_ at 37°C for 30 min. Cells were then washed again, resuspended in 0.5 ml of PBS and analyzed by FACS (Beckman Coulter) at FL-1 channel.

### Acridine Orange/Ethidium Bromide (AO/EB) Dual Staining

To determine the morphological evidence of necrosis/apoptosis in glycolipid treated cells, AO/EB staining was carried out by the method of Kasibhatla et al. (26). Briefly, cells with the density of 1 × 10^5^ were grown on 12 well plates with lysine coated glass cover slips until they form the complete monolayer. Afterward, cells were treated with various concentrations of glycolipids for 24 h and 48 h and subsequently washed with PBS as described above. Finally, treated and untreated cells were stained with acridine orange and ethidium bromide cocktail (100 µg/ml each of acridine orange and ethidium bromide mixed in PBS). Samples were examined under fluorescence microscopy (Zeiss Axio scope A1).

### Apoptosis Assay

The morphological figures of apoptotic/necrotic cells death was studied using annexin V-FITC and propidium iodide (PI) double staining apoptotic assay (Life Technology) as adapted by Rieger et al. (27). Briefly, 3 × 10^5^ cells were seeded into 6 wells and incubated for overnight. Later culture plate is treated with different concentrations of glycolipids for 48 h. Subsequently, cells were typsinized, centrifuged and pellets were washed with cold PBS. Cells were suspended in 500 µl annexin binding buffer together with 5 µl of annexin V-FITC 488 and incubated for 5 min. After the incubation, 1 µl of PI (100 µg/ml) was added to the cells and incubated for 5 min. Finally, cells were acquired by FACS (Bekman coulter) at FL-1 channel.

### Drug-Drug Interaction by Checkerboard Assay

Combinatorial effect of glycolipids was evaluated against the cancer cells by checkerboard method. Firstly, cancer cells were grown at a density of 15 × 10^3^ cells per well of 96-well plates. Secondly, serial double diluted concentration of L-SL (0–100 µg/ml) and Glucolipid (0–1000 µg/ml) were prepared in DMEM medium. Further, 10 µl of each glycolipid dilution was dispensed into 96 well plate microtiter plates. Next, glycolipid dilution made in the separate plate were transfered to plates containing cells and plates were incubated at 37°C with 5% CO_2_ for 24 h. After the incubation, 10 µl of MTT was added and plates were incubated for 3 h. Afterward, media were aspirated out and 100 µl of DMSO was added as described above and plates were read at 600 nm. The value ≤ 0.5 showed the synergistic interaction whereas value more than > 0.5 and ≤ 4 showed the additive effect.

## Results

### Effect of Glycolipids on Cell Viability

**Figure 1:**
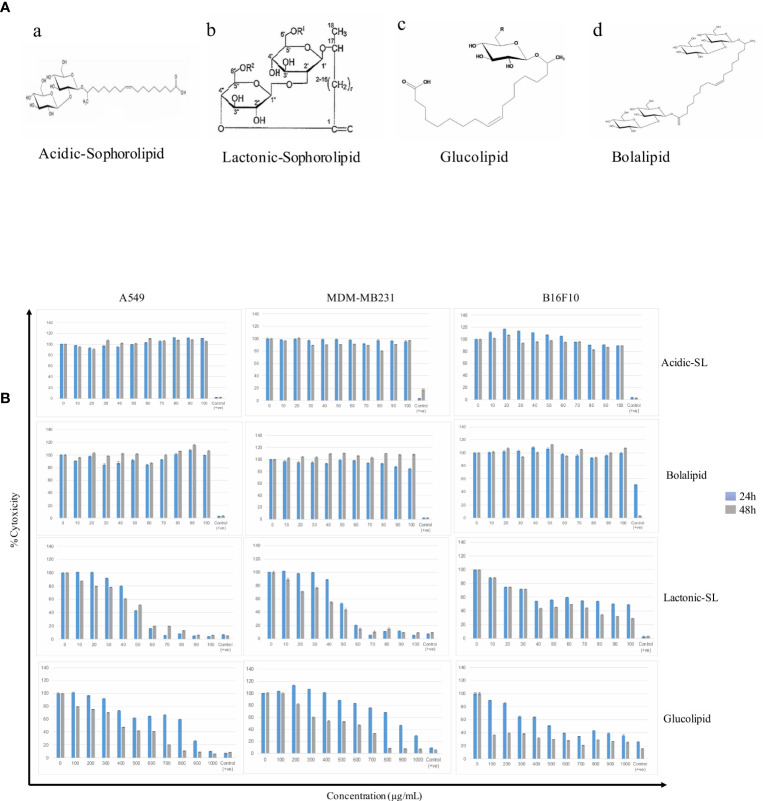
**(A)** The putative structures of different glycolipid biosurfactants used in the present study: (a) Acidic sophorolipid (A-SL); (b) Lactonic sophorolipid (L-SL); (c) Glucolipid; (d) Bolalipid **(B)**. The cytotoxic effect of glycolipid biosurfactants on different cancerous cell lines for 24 h and 48 h of treatment. The figure has been plotted in respect to DMSO vehicle control and has been done six times in three independent days, and the mean value has been shown. Statistical significance between treated and control groups was analyzed by Student's test (two-tailed, unequal variance). A p-value of <0.05 was considered statistically significant. Etoposide (10 nM) concentration was used as positive control.

A549, MDA-MB 231, and B16F10 cell lines were treated with glycolipids (acidic SL, lactonic SL, glucolipid, and bolalipid) at concentrations mentioned for 24 h and 48 h (**Figures 1A, B**). For acidic SL and bolalipid, we did not observed any change in cell viability on any cell lines used in the study (**Figure 1B**; **Supplementary Figure 1**) both after 24 h and 48 h of treatment. However for L-SL, IC_50_ values against A549, MDA-MB231, and B16F10 cell lines were 50, 50, and 40 µg/ml, respectively. Whereas, in presence of glucolipid, IC_50_ values against A549, MDA-MB 231 and B16F10, were 850, 900, and 600 µg/ml, respectively. L-SL at 100 µg/ml, completely killed A549 and MDA-MB231 cells but not the B16F10 cells. After 48 h of L-SL treatment, the IC_50_ values were reduced to 40 µg/ml for A549 and MDA-MB231, and around 35 µg/ml for B16F10. Moreover, 48 h treatment of glucolipid resulted in the reduction of IC_50_ value to 400 µg/ml against both A549 and MDM-MB231 and 100 µg/ml against B16F10. However, The effect of glycolipids on MRC-5 cells (normal fibroblast) were also evaluated (**Supplementary Figure 1**). Interestingly, we were unable to find the IC_50_ value for L-SL whereas glucolipid showed cytotoxic effects on MRC-5 cells (**Table 1**, **Supplementary Figure 1**). Furthermore, we selected lactonic-sophorolipid (L-SL, 50 µg/ml) and glucolipid (G, 500 µg/ml) concentrations in all the subsequent experiments unless stated.

**Table 1:** Toxic effects [IC_50_ (µg/ml)] of glycolipids on different cancer cell lines.

Cell lines	Lactonic -SL		Glucolipid		Acidic -SL		Bolalipid	
	24 h	48 h	24 h	48 h	24 h	48 h	24 h	48 h
A549	50	40	850	400	ND	ND	ND	ND
MDA-MB231	50	40	900	400	ND	ND	ND	ND
B16F10	40	35	600	100	ND	ND	ND	ND
MRC-5	ND	90	550	400	ND	ND	ND	ND

ND, not detected.

### Glycolipids Inhibit the Migration of Cancer Cells

**Figure 2:**
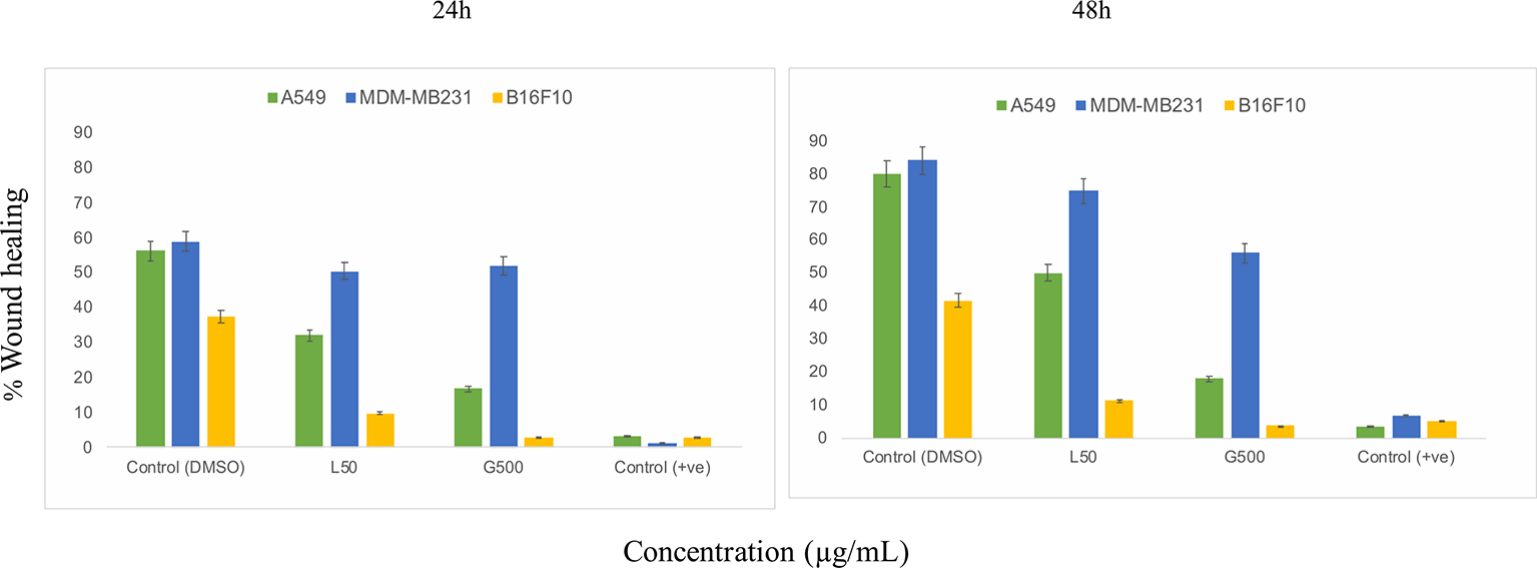
*In vitro* cancer cell migration in the presence of glycolipids after 24 h and 48 h of treatment with respect to DMSO control (0.2%, as a vehicle control). L50 (Lactonic SL 50 μg/mL) and Glucolipid (G500 μg/mL) concentration has been chosen for the experiment. Etoposide (10nM) concentration was used as a positive control. The scratch area has been measured by imageJ software. Data are presented as the average of three independent experiments ± SD. All the values are statistically significant (p<0.05) when compared with glycolipid DMSO control.

The scratch assay was done to observe the migration of the cancer cells *in vitro*. After 24 h of treatment with L-SL at 50 µg/ml (L-SL50), the relative migration of MDA-MB231, A549 and B16F10 were reduced to 84.74%, 54.54%, and 22.85%, respectively, compared to DMSO control (**Figure 2**; **Supplementary Figure 2**). Whereas, glucolipid at 500 µg/ml (G500), relative migration with respect to control were 84.74%, 27.27%, and 5.714% for MDA-MB231, A549, and B16F10, respectively. Relative migration of the cells after 48 h of treatment was also observed. In the presence of L-SL50 migration were 90.36%, 63.36%, and 25% in MDA-MB231, A549, and B16F10, respectively. Whereas, at G500 after 48 h, relative migration were observed to be 66.26%, 18.98%, and 5% for MDA-MB231, A549, and B16F10, respectively. Etoposide (25 nM) was used as a positive control.

### Effect of Glycolipids on Actin Filaments

**Figure 3:**
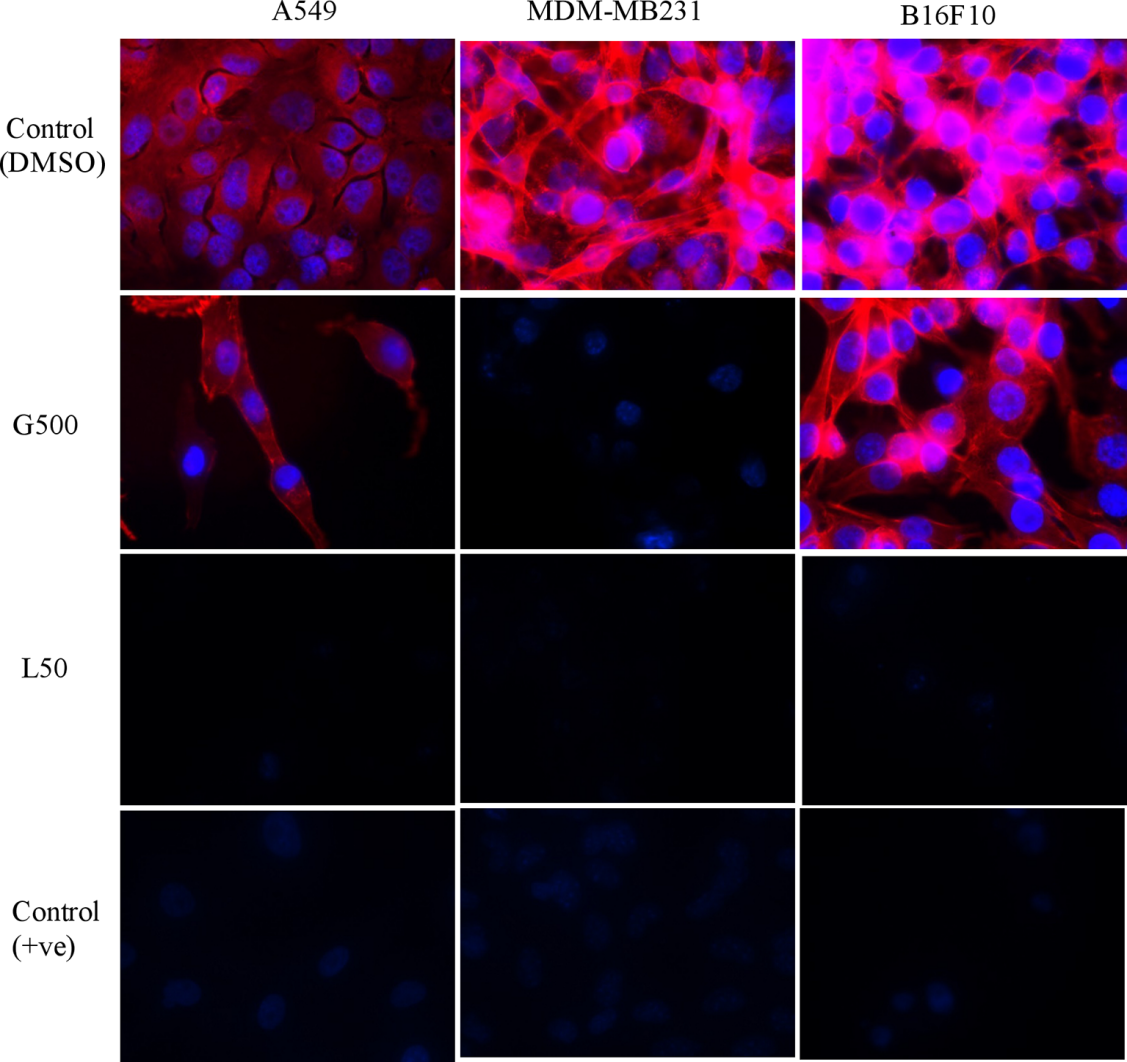
The effect of glycolipids biosurfactant on actin cytoskeleton. Cancer cells were treated with L50 (Lactonic SL 50 μg/mL) and Glucolipid (G500 μg/mL) concentration and stained with anti-phyllodin antibodies to visualize the actin filament and 4′,6-diamidino- 2-phenylindole (DAPI) to visualize nucleus. Photograph was taken at 100X magnification by flourescence microscopy. Etoposide (25nM) was used as a positive control, whereas DMSO was used as vehicle control. The experiment has been performed three times on three independent days.

The effect of glycolipids on actin filaments was tested by ZO1/phalloidin assay. Treatment with G500 and L50 has resulted in the loosening of actin filaments in A549, MDA-MB231 and B16F10 cells whereas DMSO control cells showed dense network of actin filaments attached to each other (**Figure 3**). L50 was highly effective against all the cancer cells. Treatment of G500 was highly effective against the B16F10 cells whereas MDA-MB231 showed moderate resistance toward it. No actin filaments were observed when treated with L-SL50.

### Reactive Oxygen Species Production in the Presence of Glycolipids

**Figure 4:**
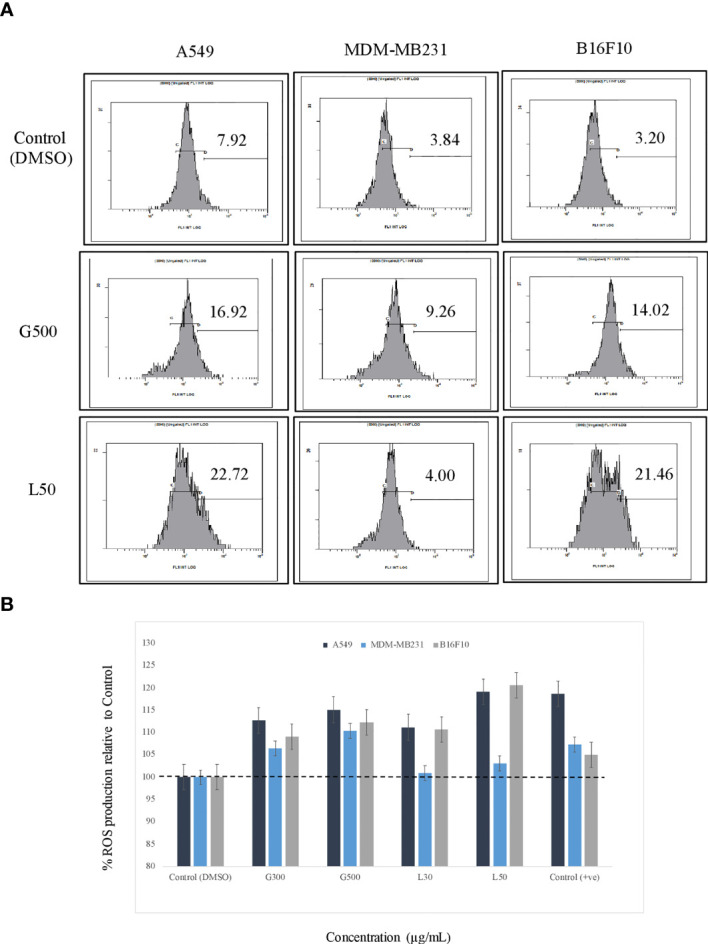
The effect of glycolipids biosurfactant on ROS (Reactive Oxygen Species) production. **(A)** Flow cytometry (FACS) analysis showing the ROS production at L50 (Lactonic SL 50 μg/mL) and Glucolipid (G500 μg/mL) concentration on different cancerous cell lines. The cells have been treated with the glycolipid for 3h and later stained with ROS specific dye H2-DCFDA. **(B)** The estimation of ROS was also measured by fluorimetric analysis, and the results have been compared with DMSO control. Etoposide was used as a positive control. The experiment has been performed three times on three independent days.

The effect of glycolipids on ROS production in the cancer cells were estimated by FACS and flurometric analysis. The result has demonstrated that after 3 h of treatment with G500 as well as L-SL50 both induced the ROS production in all the cancer cells. In the case of A549, 16.92% and 22.72% of the cells showed ROS at G500 and L-SL50, respectively. Whereas, in B16 F10 cells, 14.02% and 21.46% of the cells showed the ROS production at G500 and L-SL50. MDA-MB231 showed the negligible ROS production even at the maximum concentrations tested for glycolipids (**Figure 4A**). We also tested the ROS production by using the lesser concentrations of lactonic-SL and Glucolipid (30 µg/ml and 300 µg/ml, designated as L30 and G300, respectivily) as small doses of coumpounds are sufficient for ROS production. We observed dose dependent increase in the ROS radicals in all the cell lines used, which is also directly correlating with the FACS data (**Figures 4A, B**).

### Mitochondria Membrane Potential (MMP) Difference in the Presence of Glycolipids

**Figure 5:**
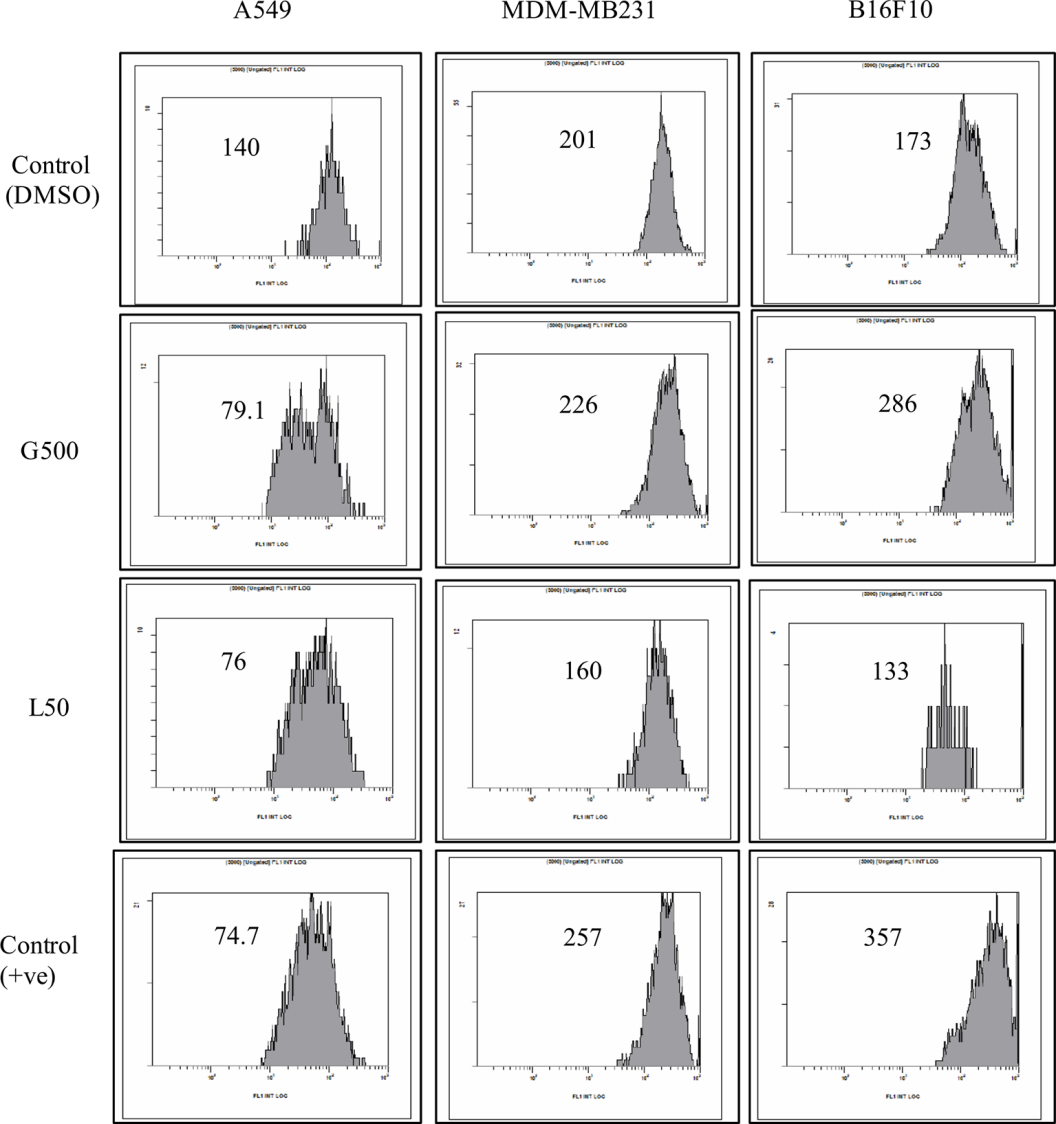
The effect of glycolipids on mitochondrial membrane potential (MMP) on different cancer cell lines. L50 (Lactonic SL 50 µg/mL) and Glucolipid (G500 μg/mL) concentration were used to study glycolipid effect on MMP change. MMP was determined by the retention of the dye 3, 3′- Dihexyloxacarbocyanine iodide (DiOC6) in cell and as measured by flow cytometry in FL-1 channel. Etoposide was used as a positive control. The experiment has been performed three times on three independent days.

The effect of glycolipids on MMP of different cancer cell lines was also evaluated. A significant difference was observed in MMP for all the tested cell lines, when treated with G500 and L-SL50. Depolarization was detected in the case for A549 cells when treated with both the glycolipids compared to DMSO control treated cells. In B16F10, hyper-polarization was observed when treated with G500 and depolarization when L-SL50 was used. MDA-MB231 cells showed little hyperpolarisation when treated with G500 and cause depolarization when treated with L-SL50 (**Figure 5**).

### Effect of Glycolipids on Cancer Cells (Live/Dead Staining)

**Figure 6:**
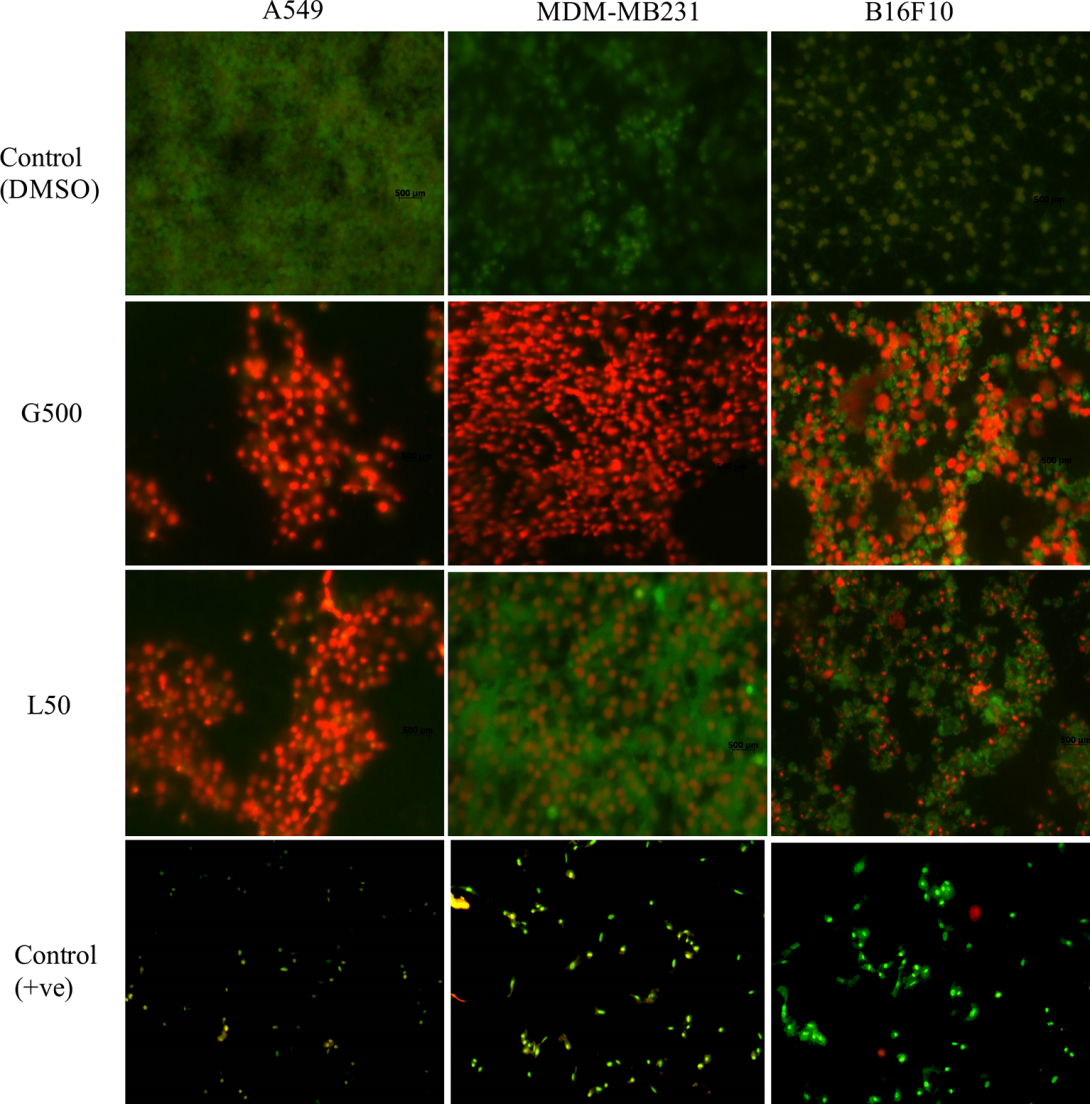
Acridine orange (AO) and ethidium bromide (EB) staining of cancerous cells after treatment of glycolipids. L50 (Lactonic SL 50 μg/mL) and Glucolipid (G500 μg/mL) concentration were used to study glycolipid effect on AO/EB staining. Photograph shows the merged images of AO and EB (60X magnification). Red spots showed necrotic bodies, whereas dark green spots showed the appearance of early stage of apoptosis. The experiment has been performed three times on three independent days.

Acridine orange and ethidium bromide (AO/EB) dual staining was used to determine apoptosis/necrosis the glycolipid treated samples. Cancer cells were treated with L-SL50 and G500 for 24 h. G500 treated MDA-MB231 cells appeared red in color indicating dead or necrosed cells. Whereas, for L-SL50 treatment, bright green spots were visualized indicating an early stage of cell death together with green viable cells. In the case of B16F10, G500, and L-SL50 treated samples both green and red colored cells were observed. Treatment of A549 cells with G500 and L-SL50 resulted in more necrotic/dead cells (**Figure 6**; **Supplementary Figure 3**).

### Glycolipid Causes Necrosis in Cancer Cells

**Figure 7:**
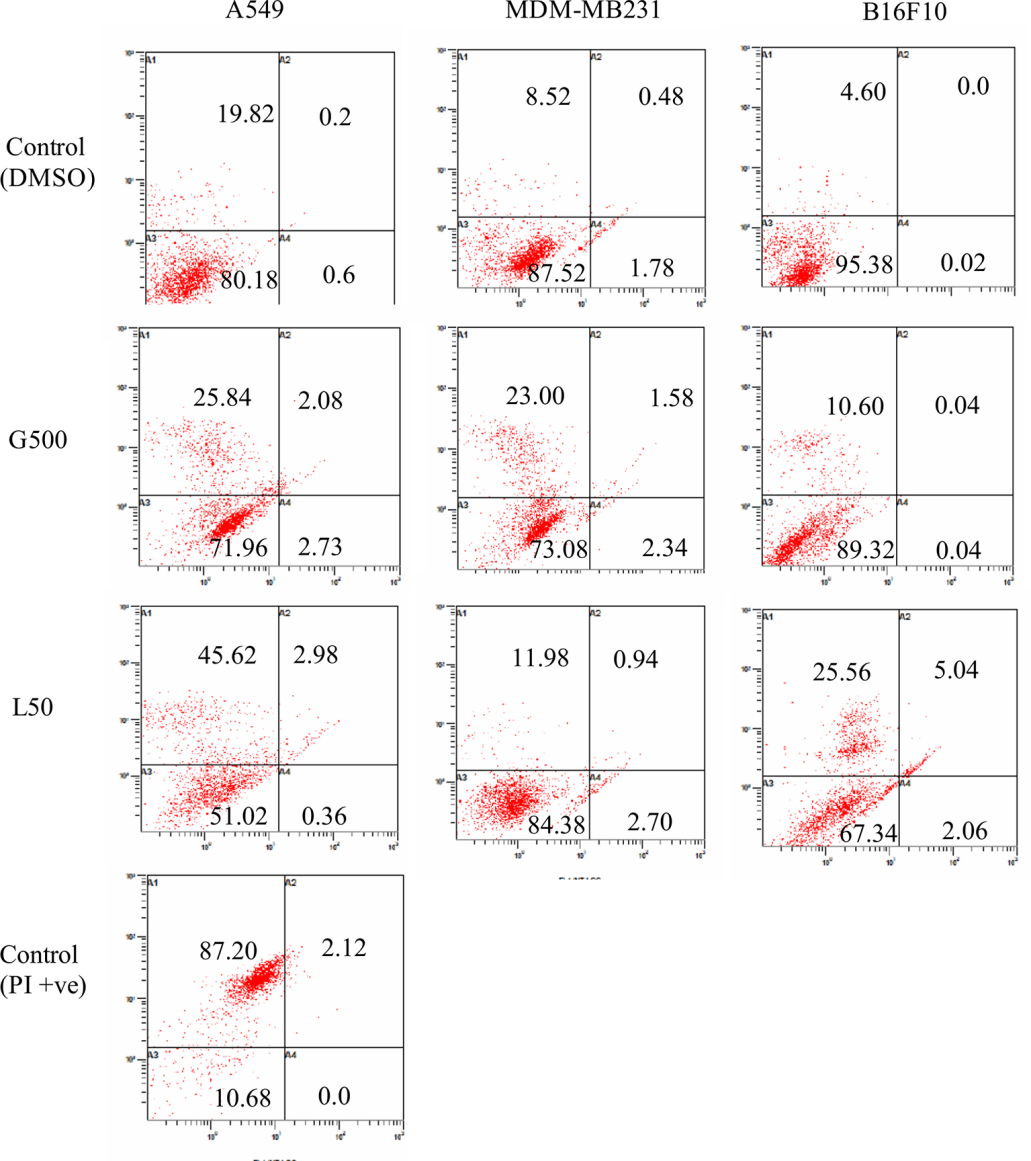
Flow cytometry (FACS) analysis of Annexin V and PI stained cancer cells after treatment with glycolipids. L50 (Lactonic SL 50 μg/ml) and Glucolipid (G500 μg/ml) concentration were used to study glycolipid effect on apoptosis/necrosis. Lower left panel of each quadrant represent untreated control; upper left panel shows the necrotic bodies, whereas upper and lower right panel showed the late and early stage of apoptosis. Samples were acquired at the FL-1 channel. For PI positive control, we heat-treated (95°C) the sample (all three cell lines, only A549 treated samples have been shown) for 15 min and later stained with Annexin V propidium iodide. The experiment has been performed three times on three independent days.

Effect of glycolipids on cancer cells was measured by FACS using Annexin/PI staining. Approximately, 26% of A549 cells, treated with G500, exhibited necrosis, however, 45% of the cells showed necrosis when treated with L-SL50. On the other hand, B16 F10 cells showed less number of necrosed cells compared to A549 cells. Only 10% and 25% populations showed necrosis when treated with G500 and L-SL50 in B16F10 cell line. MDA-MB231 was the most resistant cell line by exhibiting 11% and 23% population of the cells to be necrosed when treated with L-Sl50 and G500, respectively (**Figure 7**). Insignificant number of cell populations showed early and late stage of apoptosis in both the compounds used.

### Combinatorial Effect of Glycolipids Against the Cancer Cells

Combinatorial effect of L-SL and Glucolipid was also evaluated against all the three cancer cell lines used in this study. The predetermined IC_90_ of lactonic SL and Glucolipid against A549 were 70 and 1,000 µg/ml, respectively. Whereas, MIC_90_ of lactonic SL against MDA-MB231 was 70 µg/ml. However, MIC_90_ of Glucolipid against MDA-MB231 and B16F10 was not observed after 24 h of treatment (**Figure 1B**). Fractionation inhibitory concentration (FIC) for glycolipids (Lactonic-SL and Glucolipid) was calculated. L-SL and Glucolipid concentration of 0.25 × MIC_90_ reduced the MIC_90_ of A549 cells by 4-folds. Glucolipid at 0.125 × MIC_90_ and L-SL at 0.50 concentration × MIC_90_ reduced the MIC_90_ of MDM-MB 231 by eight and two folds, respectively. Whereas, glucolipid and L-SL at 0.25 × MIC_90_ reduced the MIC_90_ of B16F10 by 4 fold and more than 4, respectively. After the FIC calculation, Fractionation inhibitory concentration index (FICI) was calculated to determine the nature of interaction between the test compounds. L-SL and Glucolipid exhibited synergistic effect against A549 cells whereas additive effect against MDA-MB231 and B16F10 cell lines. The FICI value of L-SL and Glucolipid in combination against A549, MDA-MB231, and B16F10 were 0.5, 0.625 and ≤1, respectively (**Table 2**).

**Table 2:** The combinatorial effect of glycolipids (lactonic sophorolipid and glucolipid) on different cancer cell lines.

Cell lines	FIC		FICI
A 549	Glu 0.25	SL 0.25	0.5
MDA-MB231	GlU 0.125	SL 0.5	0.625
B16F10	Glu 0.5	SL 0.5	1

## Discussion

In spite of advancements achieved in combating cancers, resistance to classical chemotherapeutic agents continue to be a major obstacle in cancer therapies and accountable for most relapses, one of the major causes of death in cancer (28, 29). Moreover, the effectiveness has often been limited by toxicities on normal tissues/cells, poor solubility and stability, and limited bio-distribution (30, 31). Thus, it is vital to create alternative efficient formulations that can address the above mentioned challenges and provide selective targeting of cancer cells without significant damage to the viability of healthy cells.

Interest in biosurfactants has been increased from past few years due to their physiochemical as well as biomedical properties (32). Glycolipids BSs have great impact in the field of medical sciences and proven to have better effects than other BSs (4). Rhamnolipid, Sophorolipid, mannosylerythritol lipid with other glycolipids have been reported to possess antimicrobial, anticancer, and antiadhesive properties (3, 7, 33). Therefore, we attempted to evaluate anticancer potential of certain glycolipids of natural origin as shown in **Figure 1A**, i.e., bolalipid, glucolipid, acidic-sophorolipid, and lactonic-sophorolipid and explored their possible mode of action in cancer cell. In the present study, lactonic-sophorolipid (L-SL) and Glucolipid exerted cytotoxic effects against the cancer cell lines (A549, MDM-MB 231, and B16F10) but bolalipid (Bola) and acidic-sophorolipid (A-SL) did not show any effect. Shao et al. (10) have also reported non-cytotoxicity of A-SL on esophageal cancer cells (11). Bolalipid is one of the class of bolaamphiphilic compounds having two sophorose sugars attached at both ends of the lipid chain (34). On the other hand, there is no report on cell cytotoxicity using bolalipids *in vitro* as well as *in vivo*, makes them advantageous to be used in drug/gene delivery nano-carriers (34, 35).

Out of the four tested glycolipids, L-SL has shown better effects followed by glucolipid. A similar report for L-SL, produced by *Wickerhamiella domercqiae*, on different cancer cell lines has been published (9). Nevertheless, no anticancer activity of glucolipid is reported in the literature. In our study, the IC_50_ of glucolipid is much higher than the L-SL in all the cell lines used. Moreover, complete killing of the cells was not observed for glucolipid even at the highest concentration used. The degree of effectiveness of glycolipids is thought to be due to the presence of different head groups attached to the lipid chain. Shah et al. (32) observed enhanced antibacterial activity of L-SL when the cells were grown in different carbohydrate containing media (7). Cell viability of control human fibroblast cells (MRC-5) was also checked in the presence of glycolipids (**Supplementary Figure 1**). For L-SL However, moderate cytotoxiciy was found at the highest concentration (100 µg/ml) of L-SL used after a treatment of 24 h. After 48 h of treatment, IC_50_ value was 90 µg/ml for MRC5 cells whereas at this concentration, only 10% cell viability was observed for all other cancer cells except B16F10 (i.e., 30% viability). For glucolipid, IC_50_ value against MRC-5 was 550 µg/ml and 400 µg/ml for 24 h and 48 h of treatment, respectively and considered to be toxic for normal cells whereas L-SL was safe. Findings from other researachers also suggested the safe use of lactonic-SL (10).

Cancer cell posses a broad range of migration and invasion mechanisms (36). In recent time, it has led therapeutics against the cancer cells progression to be failed in clinical trials (7). Therefore, we also investigated the effect of L-SL and glucolipid on A549, MDA-MB231 and B16 F10 cell migration *in vitro*. The result showed that both compounds effectively inhibited the cell migration after 24 h and 48 h of treatments. MDA-MB231 showed highest resistance followed by A549 and B16F10. Huang et al. (37) have reported that brazilein, a compound isolated from *Caesalpinia sappan L*., inhibited the migration of MDA-MB231 cell by inhibiting the expression of matrix metalloproteinase-2 (MMP-2). Matrix metalloproteinase is considered to play a crucial role in the cell migration in other cancer cells (37, 38). Another important factor for cell migration is attributed to actin cytoskeleton, which are upregulated in invasive and metastasis cancer cells (39). We observed that L-SL and Glucolipid disrupted the existing network of actin filaments and thereby inhibited the cell migration. Similarly, Senderowicz et al. (40) reported that jasplakinolide, a cyclodepsipeptide, inhibited the migration of PC3 cells by disrupting actin cytoskeleton. The actin cytoskeleton is a physiological regulator of ROS release from mitochondria, and a key element in the upstream activation of cell death pathways (41). ROS are formed as a natural by-product of the normal metabolism of oxygen and have an important role in celluler homeostasis and signaling, but during the stress condition, the level of ROS increases dramatically. Therefore, we estimated the production of ROS in the presence of glycolipids using H_2_-DCFDA dye. DCFH-DA can easily pass through the cell membrane and is de-esterified by intracellular esterases to the nonfluorescent polar derivative DCFH, which is oxidized to highly fluorescent dichlorofluorescein (DCF) in the presence of ROS. Interestingly, ROS was produced by all the cancer cells used, except MDA-MB 231. We speculate that it could be due to its higher resistance. L-SL induced more ROS production compared to the glucolipid. Similar findings for ROS production in the presence of L-SL on breast cancer cell line, were reported by Ribeiro et al. (42). It has been reported in the literature that mitochondria are the vital source for the ROS production and cellular homeostasis (43). This homeostasis is changed during the stress conditions in the form of change in the MMP. Therefore, we measured the MMP of cell lines in the presence of glycolipids, and found a significant change in the potential difference when compared to the vehicle control (DMSO).

Furthermore, the mechanism of cell death was further explained by using acridine orange and ethidium bromide dual staining and flow cytometry experiments. Acridine orange is a green vital dye which stains the live and dead cells and emits green florescence when binds to the double helix, whereas ethidium bromide stains only dead cells and emits red florescence. Early apoptotic bodies appear as bright green spots with condensed or fragmented chromatin and late apoptotic bodies display condensed and fragmented orange nucleus (44). We found that both L-SL and Glucolipid caused cell death in all the three cell lines. Most of the cells appeared red, however, a few cells showed the bright green spots, which indicated that the cells underwent necrosis. Moreover, to assure necrosis dependent killing, we further examined it by flow cytometry experiments using Annexin/PI staining. During the apoptosis, the membrane phospholipid phosphatidylserine (PS) is translocated from inner to the outer leaflet of the plasma membrane. Annexin has the capability to bind the surface exposed PS and give positive signals whereas PI only enters to the dead or damaged cells and excluded by the healthy/live cells. We observed high percentage of cell population appeared to be PI positive, which has indicated the necrosis in the cells. There are various treatments used for anticancer therapy which also may cause necrosis in tumor cells (45). Outcomes of our study showed ROS-dependent necrosis in the cancer cells. Some reports also suggested possible role of kinase receptor-interacting protein (RIP1) in ROS-dependent necrosis (46, 47). We hypothesize that in the present study, it might be possible that L-SL and Glucolipid could induce ROS-dependent necrosis *via* activation of RIP1 and causes cell death in cancer cells.

Many conventional chemotherapeutic anticancer agents kill cancer cells by directly damaging their DNA, which has the problem of non-specificity and relatively high toxicity (29). In this regard, combinatorial therapy is considered best to reduce the toxicity and increase the efficacy of the compounds (48). The combination of non-steroidal inflammatory drugs was shown to act synergistically in the ovarian cancer cell line (49). In the present study, we examined the combinatorial effect of L-SL and Glucolipid on lung (A549), breast cancer (MDA-MB231) and mouse melanoma (B16F10) cell lines. Interestingly, both the compounds act synergistically against A549 cells as 4-folds reductions were observed. However, additive effects were seen against B16F10 and MDA-MB231 cell lines as they reduced the IC_90_ value by 2-folds only. Synergistic interaction of curcurbitacin B with other chemotherapeutic drugs was also reported on A549 cells and proven better than the available drugs (50). Similar results for synergistic interaction on triple negative breast cell line by rapamycin and lapatinib has also been reported by Liu et al. (51). These two glycolipids interacted synergistically/additively on the cancer lines, therefore, could be used at very low concentration to avoid normal cell toxicity, and with increased efficacy on cancer cells.

In recent decades, more and more targeted drugs have been used to specifically target/prevent changes that push cancer growth and spread. However, initial outcomes of these drugs are quite effective, a large number of patients develop resistance as medication progresses (29). For example, 30%–55% of patients with non-small cell lung cancer (NSCLC) relapse and die from the disease subsequently (52). The 50%–70% of ovarian adenocarcinomas reoccur within 1 year post-surgery and associated chemotherapy (53). The multiple targets in combination would tackle the problem of emerging drug resistance and relapses. Furthermore, poor pharmacokinetic characteristics of anticancer drugs could also be overcome as these biosurfactants have been proven to show easy bioavailability and drug delivery (1, 4, 5, 28, 29, 54, 55). However, the pathways for glycolipids induced necrosis need to be further elucidated. Present findings suggest that lactonic-sophorolipid and glucolipid could be a valuable addition to search for novel anticancer therapeutics.

### Statistical Evaluation

All experiments were performed in triplicate and on three different days. All data were expressed as mean values with the corresponding standard deviations (SD). Statistical significance between treated and control groups was analyzed by Student’s t-test (two-tailed, unequal variance). A p-value of <0.05 was considered statistically significant.

## Data Availability Statement

The original contributions presented in the study are included in the article/**Supplementary Material**. Further inquiries can be directed to the corresponding authors.

## Author Contributions

FH designed and performed the experiments and wrote the manuscript. MSAK analyzed and reviewed the data, and revised the manuscript. All authors contributed to the article and approved the submitted version.

## Conflict of Interest

The authors declare that the research was conducted in the absence of any commercial or financial relationships that could be construed as a potential conflict of interest.
