# Supplementary material for: ROS-Mediated Necrosis by Glycolipid Biosurfactants on Lung, Breast, and Skin Melanoma Cells
Source: Front Oncol. 2021 Mar 16;11:622470. doi: 10.3389/fonc.2021.622470 (PMC8009627; doi:10.3389/fonc.2021.622470)
Supplement: Supplementary file 1 [file Presentation_1.pptx]

## Slide 1
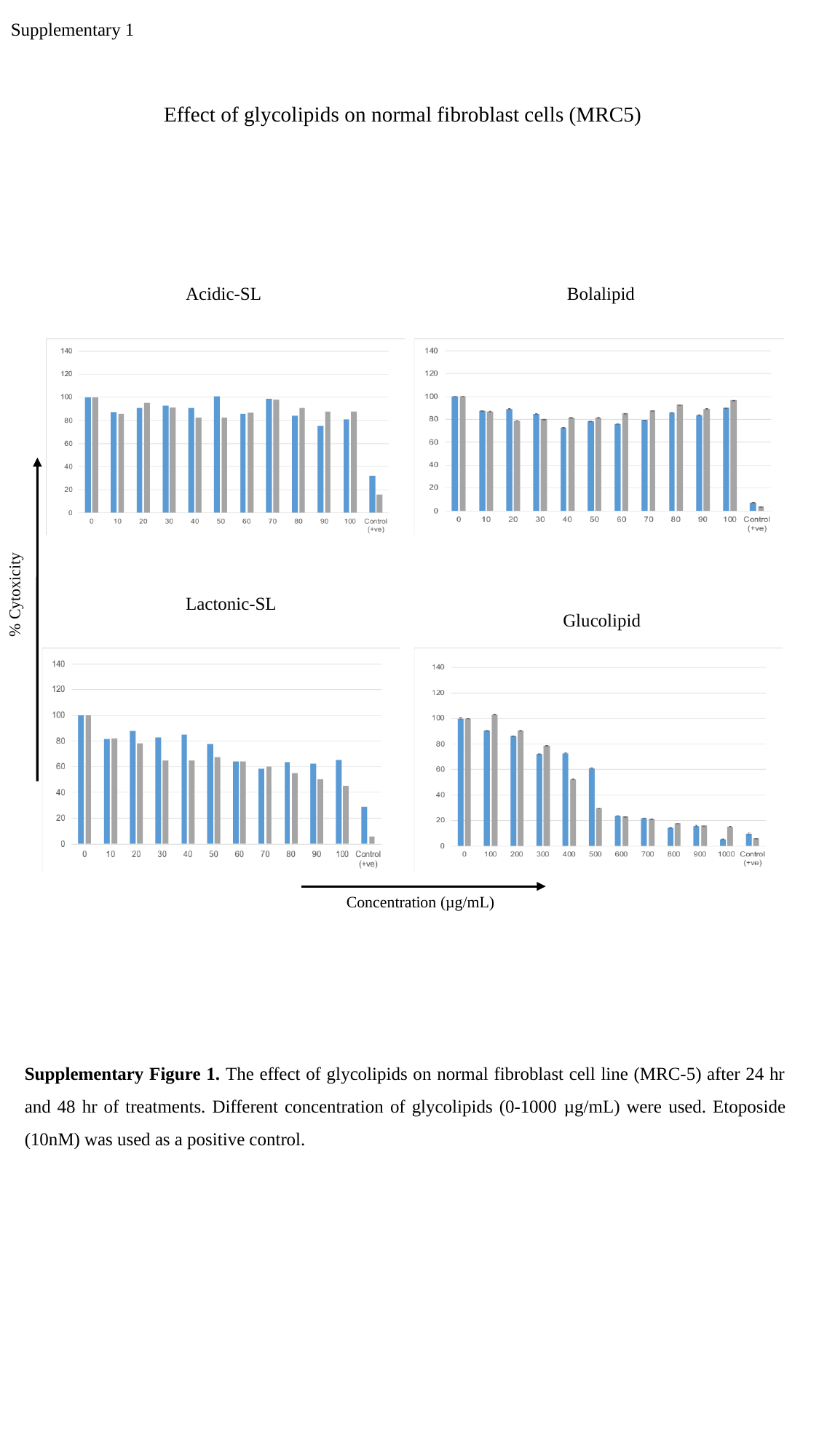

Supplementary 1
Effect of glycolipids on normal fibroblast cells (MRC5)
Acidic-SL
Bolalipid
% Cytoxicity
Lactonic-SL
Glucolipid
Concentration (µg/mL)
Supplementary Figure 1. The effect of glycolipids on normal fibroblast cell line (MRC-5) after 24 hr and 48 hr of treatments. Different concentration of glycolipids (0-1000 µg/mL) were used. Etoposide (10nM) was used as a positive control.

## Slide 2
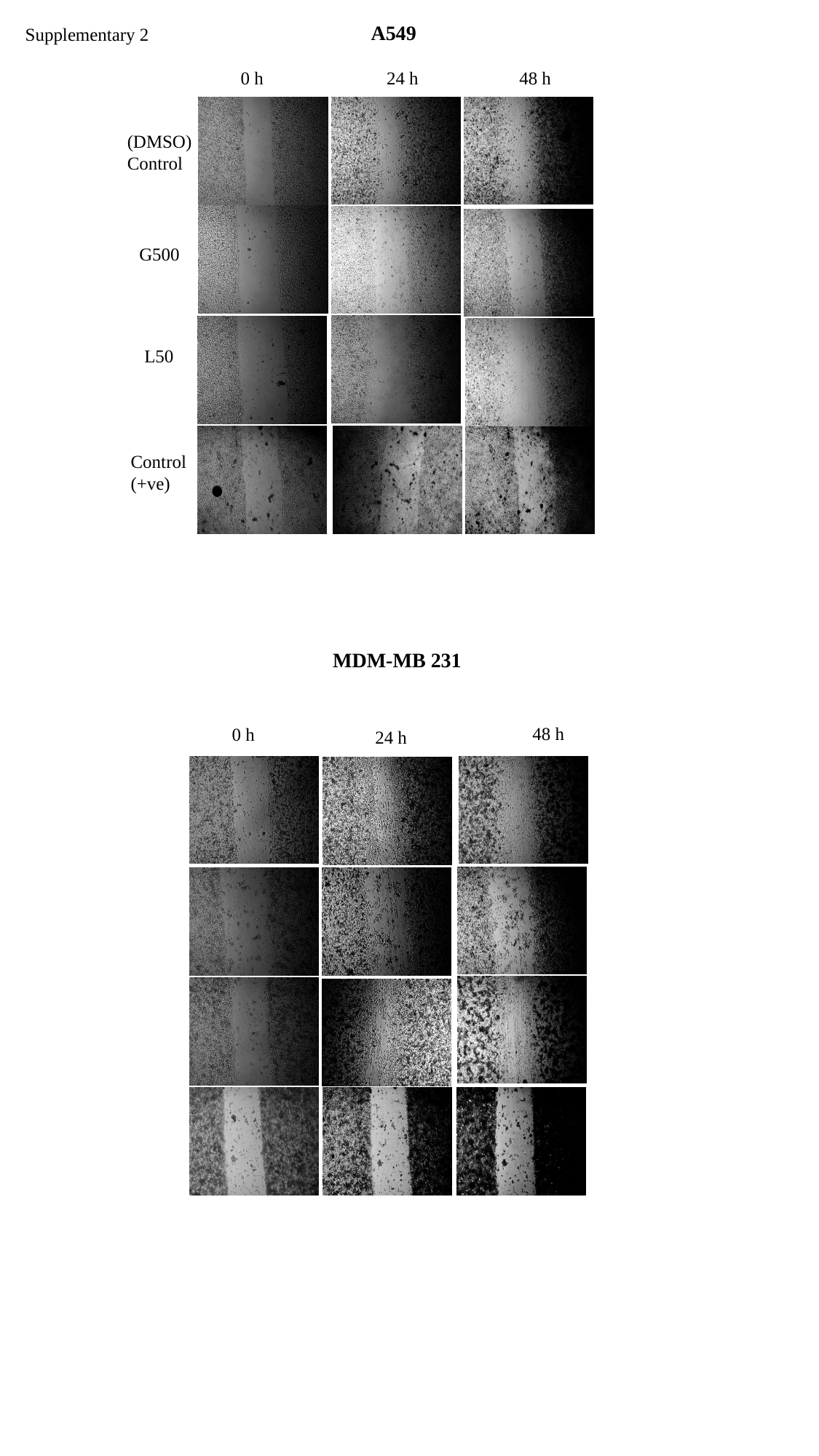

A549
Supplementary 2
0 h
24 h
48 h
(DMSO)
Control
G500
L50
Control
(+ve)
MDM-MB 231
48 h
0 h
24 h

## Slide 3
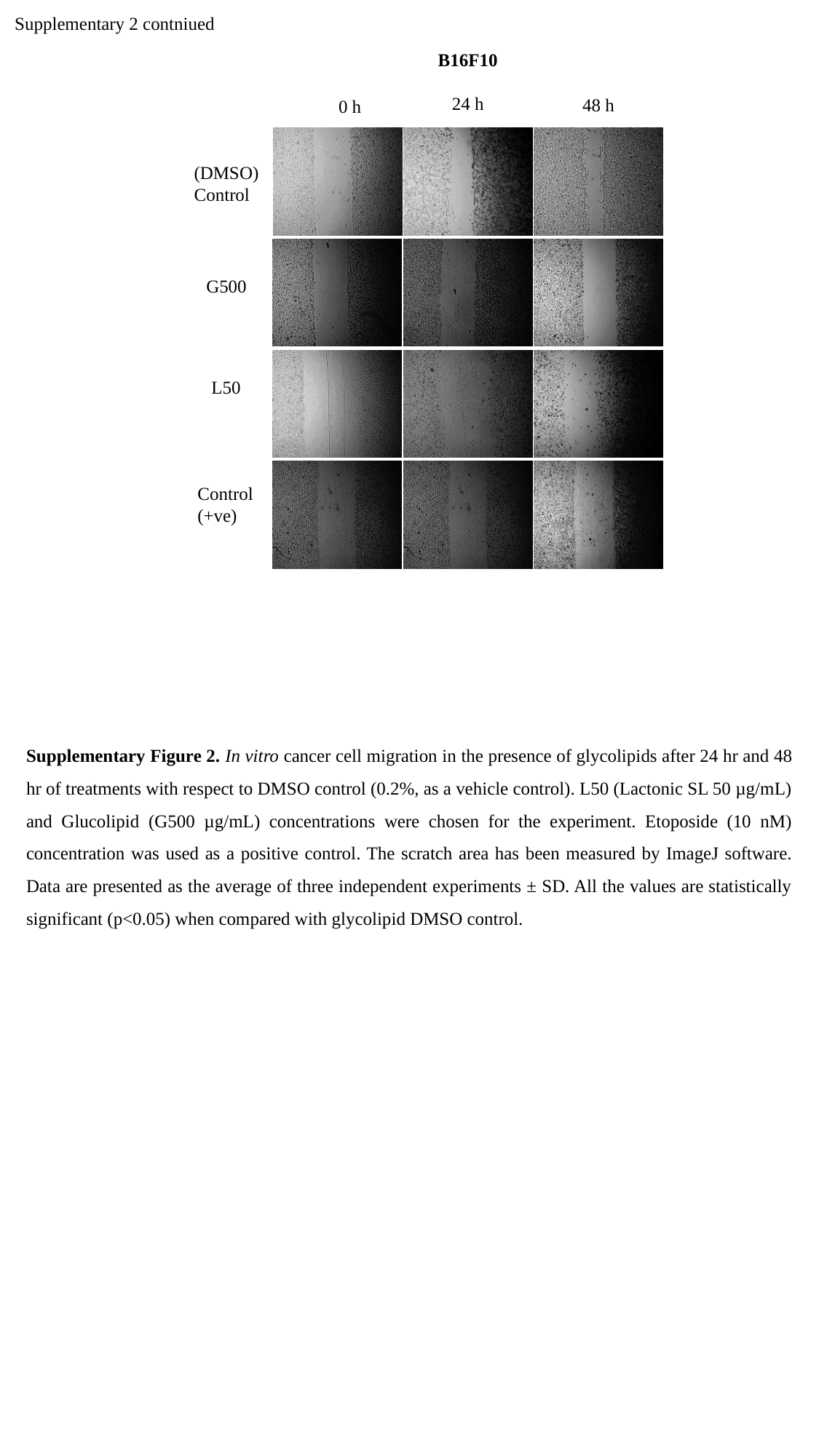

Supplementary 2 contniued
B16F10
24 h
48 h
0 h
(DMSO)
Control
G500
L50
Control
(+ve)
Supplementary Figure 2. In vitro cancer cell migration in the presence of glycolipids after 24 hr and 48 hr of treatments with respect to DMSO control (0.2%, as a vehicle control). L50 (Lactonic SL 50 µg/mL) and Glucolipid (G500 µg/mL) concentrations were chosen for the experiment. Etoposide (10 nM) concentration was used as a positive control. The scratch area has been measured by ImageJ software. Data are presented as the average of three independent experiments ± SD. All the values are statistically significant (p<0.05) when compared with glycolipid DMSO control.

## Slide 4
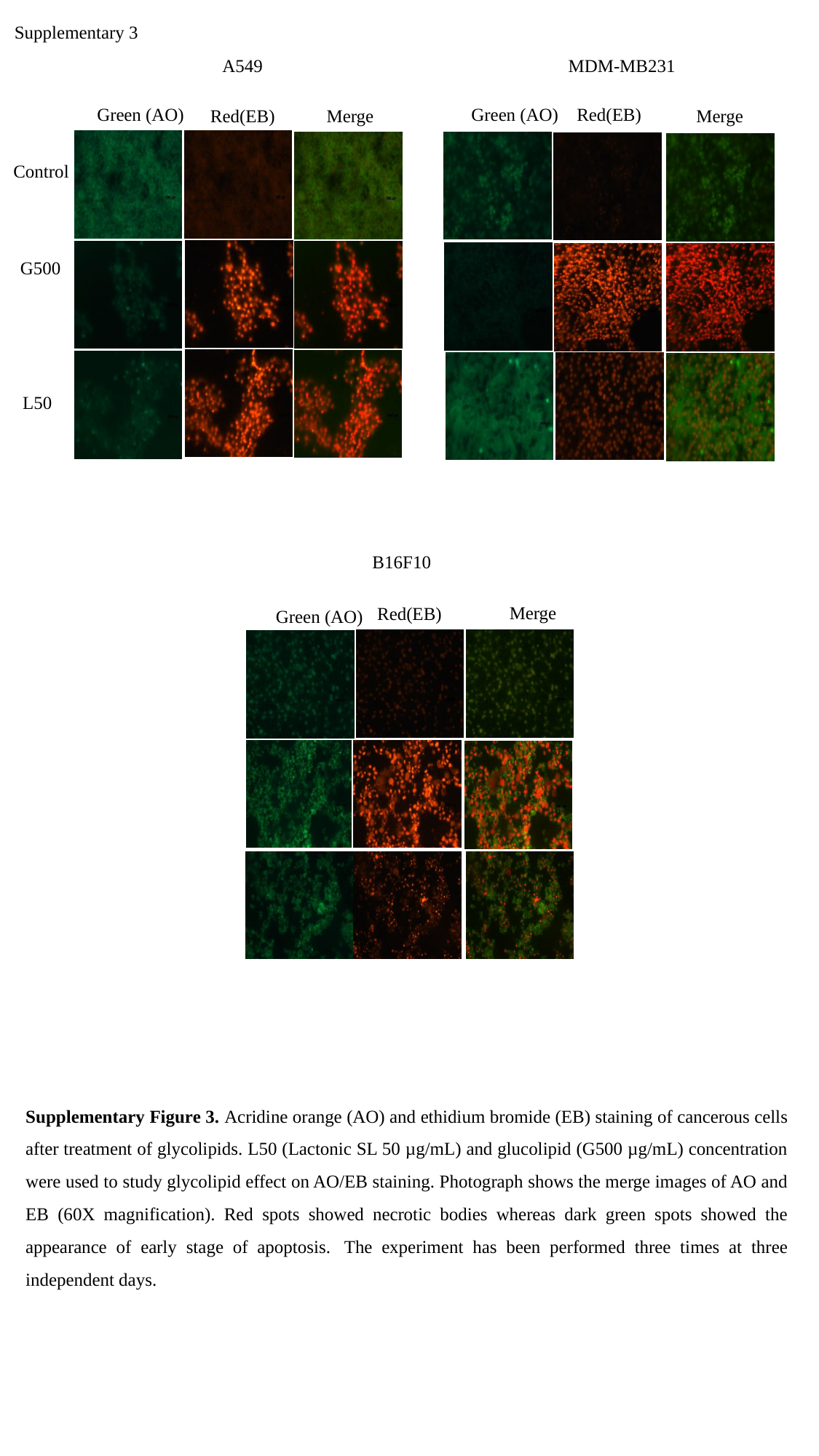

Supplementary 3
A549
MDM-MB231
Green (AO)
Red(EB)
Green (AO)
Red(EB)
Merge
Merge
Control
G500
L50
B16F10
Merge
Red(EB)
Green (AO)
Supplementary Figure 3. Acridine orange (AO) and ethidium bromide (EB) staining of cancerous cells after treatment of glycolipids. L50 (Lactonic SL 50 µg/mL) and glucolipid (G500 µg/mL) concentration were used to study glycolipid effect on AO/EB staining. Photograph shows the merge images of AO and EB (60X magnification). Red spots showed necrotic bodies whereas dark green spots showed the appearance of early stage of apoptosis.  The experiment has been performed three times at three independent days.
